# The Late Sir Andrew Clark

**Published:** 1900-03-24

**Authors:** 


### EDITOR'S LETTER-BOX.

[Our Correspondents are reminded that prolixity is a great bar to publication, and that brevity of style and conciseness of statement greatly facilitate early insertion.]

### THE LATE SIR ANDREW CLARK.

"Alpha" writes: A few months after the death of the late Sir Andrew Clark, M.D., persons who had had correspondence with him were invited to send any letters from him which they had in order to facilitate the writing of his life. This occurred six and a-half years since, and we hear nothing further about the book. Sir Andrew was on very intimate terms with many people highly placed, both in the social and political worlds. Is the book being "burked" because it would contain matter distasteful to these people or their families? If so, why does not someone issue the medical part of the work by itself, and, as in the case of "Talleyrand's Memoires," the family defer the publication of the other portion until all those whom it might affect have joined the majority?

